# Toxins turned into medicines: A systematic review of peptide animal-derived venom in wound treatments

**DOI:** 10.1016/j.cjtee.2025.08.006

**Published:** 2026-04-25

**Authors:** Alessandra Silva Teles, Thais Lima, Andréa Gonçalves de Melo, William Gustavo Lima, Lívia Cristina de Paiva Gardoni, Israel Júnior Borges do Nascimento, Julio Cesar Moreira Brito

**Affiliations:** a Universidade Federal de Minas Gerais, Belo Horizonte, Minas Gerais, Brazil; b Fundação Ezequiel Dias, Belo Horizonte, Minas Gerais, Brazil; c Instituto de Ensino e Pesquisa, Santa Casa, Belo Horizonte, Minas Gerais, Brazil; d Division of Country Health Policies and Systems, World Health Organization, Regional Office for Europe, Copenhagen, 2100, Denmark

**Keywords:** Peptides, Wound treatments, Toxins, Healing

## Abstract

**Purpose:**

Venoms derived from animals, including their fractions, toxins, and isolated compounds, have been extensively studied as potential sources for drug development. However, their inherent toxicity and limited plasma stability pose a major challenge for parenteral administration. Despite these obstacles, clinical research on these compounds for wound healing has shown positive results and attracted increasing attention in recent years. Therefore, this systematic review aims to comprehensively evaluate the efficacy, safety, and current clinical research status of toxins and related peptides in wound treatment.

**Methods:**

A systematic review was conducted from database inception to July 7, 2025 and included 4 databases: PubMed/MEDLINE, Scopus, Embase, and LILACS. Search terms included “peptides”, “toxins”, “injuries”, and “*in vivo*” to ensure broad inclusion criteria for the target population. Two independent researchers performed study screening and data extraction, with a discussion group responsible for resolving any discrepancies. The data were critically analysed and descriptively summarised in the manuscript.

**Results:**

After the database search, 19 studies were classified as eligible based on the predefined inclusion and exclusion criteria. The analysis identified peptides derived from the venoms of various animal species, including snakes, spiders, scorpions, lizards and wasps. These peptides exhibited diverse mechanisms of action, biological functions and therapeutic applications, which emphasises their high complexity.

**Conclusion:**

These results suggest that further research on the development of novel biopharmaceuticals from animal venoms for topical application is highly promising, especially for the treatment of hard-to-heal wounds.

## 1 Introduction

Wound healing is a sequence of cellular and molecular events aimed at restoring tissue integrity after injury. This biological process is classically delineated into 4 overlapping phases: hemostasis, inflammation, proliferation, and remodelling.^1^^,^^2^ Immediately following tissue damage, hemostasis is initiated to halt blood loss. This phase involves localised vasoconstriction, platelet activation, and ultimately culminates in the formation of a hemostatic plug and a fibrin clot.^1^ Concurrently, the inflammatory phase commences, characterised by vasodilation, increased vascular permeability, and the recruitment of immune cells, such as neutrophils and macrophages, to the injury site.^1^^,^^3^ Subsequently, the proliferative phase is marked by the generation of new tissue to bridge the discontinuity and close the wound.^2^ Finally, the remodelling phase, the longest of the 4 phases, involves the maturation and organization of newly synthesised collagen, which imparts strength and elasticity to the scar tissue.^4^

Various compounds can modulate and support each of these healing stages. Among these, peptides derived from animal toxins have been studied for their diverse biological activities across multiple phases of wound repair.^5^^,^^6^ Animal toxins are complex mixtures of bioactive substances, predominantly proteins and peptides, originating from various animal groups. These molecules, products of secondary metabolism, are capable of eliciting deleterious effects in other living organisms by disrupting essential physiological functions.^7^ They play crucial roles in predation, defence, interspecies competition, and even intraspecies communication.^7^

However, peptides isolated from animal toxins represent an immense and diverse range of biomolecules with unique characteristics that depend on the animal species from which the toxin was extracted. Therefore, despite their therapeutic potential, these peptides often exhibit toxic effects that must be thoroughly assessed before clinical application.^5^^,^^6^

Therefore, this systematic review aims to comprehensively evaluate the applicability, efficacy, safety, and current state of knowledge regarding animal-derived venom peptides in wound treatment and healing.

## 2 Methods

Our systematic review was conducted in accordance with the recommendations of the Cochrane Handbook.^8^ In addition, we strictly adhered to the PRISMA (Preferred Reporting Items for Systematic Reviews and Meta-analyses) statement when searching for, selecting, extracting, and analyzing relevant data.

### 2.1 Search strategy and selection criteria

The systematic review, which ran until July 7, 2025, initially searched 4 different databases (Embase, LILACS, PubMed/MEDLINE, and Scopus). Briefly, the search strategy included terms related to “peptide”, “polypeptide”, “toxins”, “injury”, “sore”, “injured”, “damage”, “damaged”, “bruised”, “crushed”, “scarring”, “animal”, “rat”, “mice”, “murine”, “rabbit”, and “*in vivo*”. In addition, the search was expanded to include the corresponding MeSH descriptors. The reference lists of all included research papers and relevant reviews were screened to minimise the risk of missing potentially important data.

Studies were included if they evaluated the cicatrization potential (healing properties) of peptide derivatives derived from vertebrate or invertebrate toxins. When analyzing potential healing effects, we considered only *in vivo* studies employing unbiased assessment methods. Assessment methods included histological analysis (e.g., assessment of tissue regeneration, collagen deposition, and inflammation), biochemical analysis (e.g., quantification of growth factors, cytokines, or matrix metalloproteinases), mechanical testing (e.g., assessment of tensile strength or wound closure), and imaging techniques (e.g., ultrasound and computed tomography, etc.).

Review articles, notes, editorials, letters, communications, or articles whose full texts were not available, or that did not contain relevant data relevant to our research question, were excluded. Apart from the use of the venom in kind (crude venom), other exclusion criteria included: (1) no definition of the specific peptide used; (2) use of exclusively *in vitro* or in silico studies that only evaluated peptide interaction with a molecular target (in silico studies); and (3) studies that evaluated the curative activity of proteins, enzymes, polysaccharides or other non-peptidic compounds isolated from animal venoms.

### 2.2 Study selection process

The databases were searched by 2 different researchers (Alessandra Silva Teles and Thais Lima). The titles and abstracts were screened against the eligibility criteria, and duplicate entries were removed. The full-text articles that evaluated the efficacy of animal venom-derived peptides in wound healing were then selected.

The following characteristics were considered relevant in the articles that met the inclusion criteria: peptide information (name, number of residues, and source species), wound type, active concentration, and toxic concentration. Each phase of data extraction was performed by at least 2 different researchers (Alessandra Silva Teles and Thais Lima). Discrepancies were clarified through dialogue to prevent inaccurate information extraction, and the risk of bias was assessed using the SYRCLE Risk of Bias (RoB) tool.^9^

### 2.3 Quality assessment

The level of agreement between reviewers was analyzed using Cohen's kappa coefficient, with a 95% confidence interval (*CI*).^10^ Any disagreements were resolved through discussion with a third reviewer (Julio Cesar Moreira Brito).

## 3 Results

### 3.1 Literature search

A search of the databases yielded 3045 publications, as shown in Fig. 1 (587 from Embase, 226 from LILACS, 225 from PubMed/MEDLINE, and 2007 from Scopus).^8^ After excluding duplicates and adding records identified through other sources, 1501 studies were screened to ensure that the titles, abstracts, and keywords met the inclusion criteria. During the thorough evaluation of the remaining 26 studies, 7 were disqualified, 4 because the study focused on crude venom, 2 because the full article was not available, and 1 because the article did not fit the pre-established design for the review (Supplementary Data).^5^^,^^11^, ^12^, ^13^, ^14^, ^15^, ^16^, ^17^, ^18^, ^19^, ^20^, ^21^, ^22^, ^23^, ^24^, ^25^, ^26^, ^27^, ^28^

**Fig. 1:**
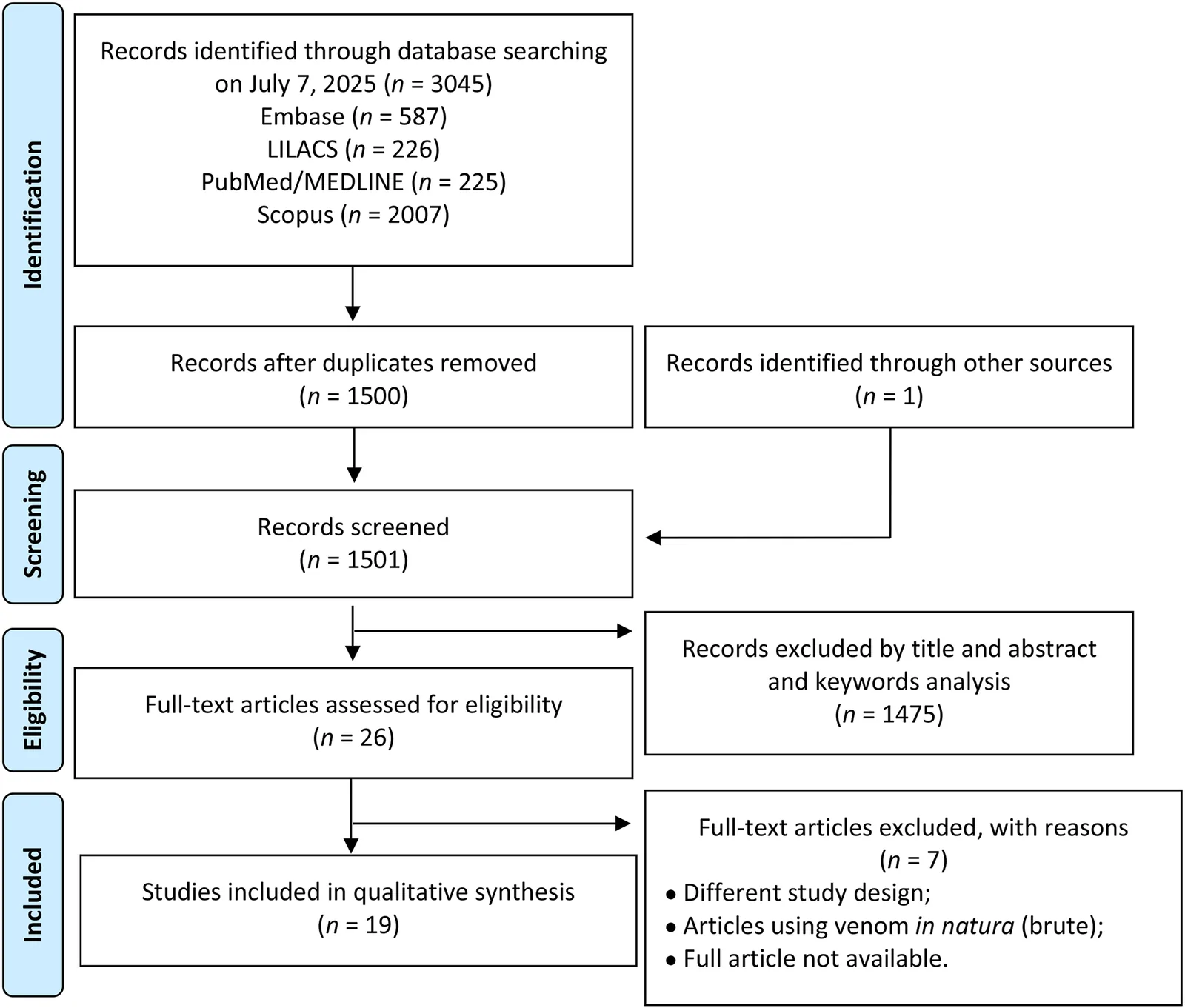
Flowchart of the article selection for systematic review and meta-analysis according to PRISMA criteria.^8^

After a detailed analysis of the remaining 19 studies,^5^^,^^11^, ^12^, ^13^, ^14^, ^15^, ^16^, ^17^, ^18^, ^19^, ^20^, ^21^, ^22^, ^23^, ^24^, ^25^, ^26^, ^27^, ^28^ the relevant data were collected and further processed. The degree of agreement between the 2 authors who researched and selected the articles in the inter-rater reliability test was almost perfect, as shown by the Kappa concordance index (Kappa of 0.887).^8^

Regarding the origin of the peptides, 7 studies used peptides derived from arthropod venom^19^, ^20^, ^21^^,^^23^^,^^25^, ^26^, ^27^ (7/19, 36.8%). Of these 7, 3 were isolated from scorpions (3/19, 15.8%),^19^^,^^21^^,^^23^ 1 from a bee (1/19, 5.3%),^20^ 2 from wasps (2/19, 10.5%),^25^^,^^26^ and 1 from a spider (1/19, 5.3%).^27^ Eight studies used snake venom (8/19, 42.1%),^5^^,^^11^^,^^14^, ^15^, ^16^^,^^22^^,^^24^^,^^28^ 1 used a peptide derived from frog venom (1/19, 5.3%),^13^ and 3 studies used exendin-4, a peptide derived from lizard venom (3/19, 15.8%),^12^^,^^17^^,^^18^ as shown in Fig. 2.

**Fig. 2:**
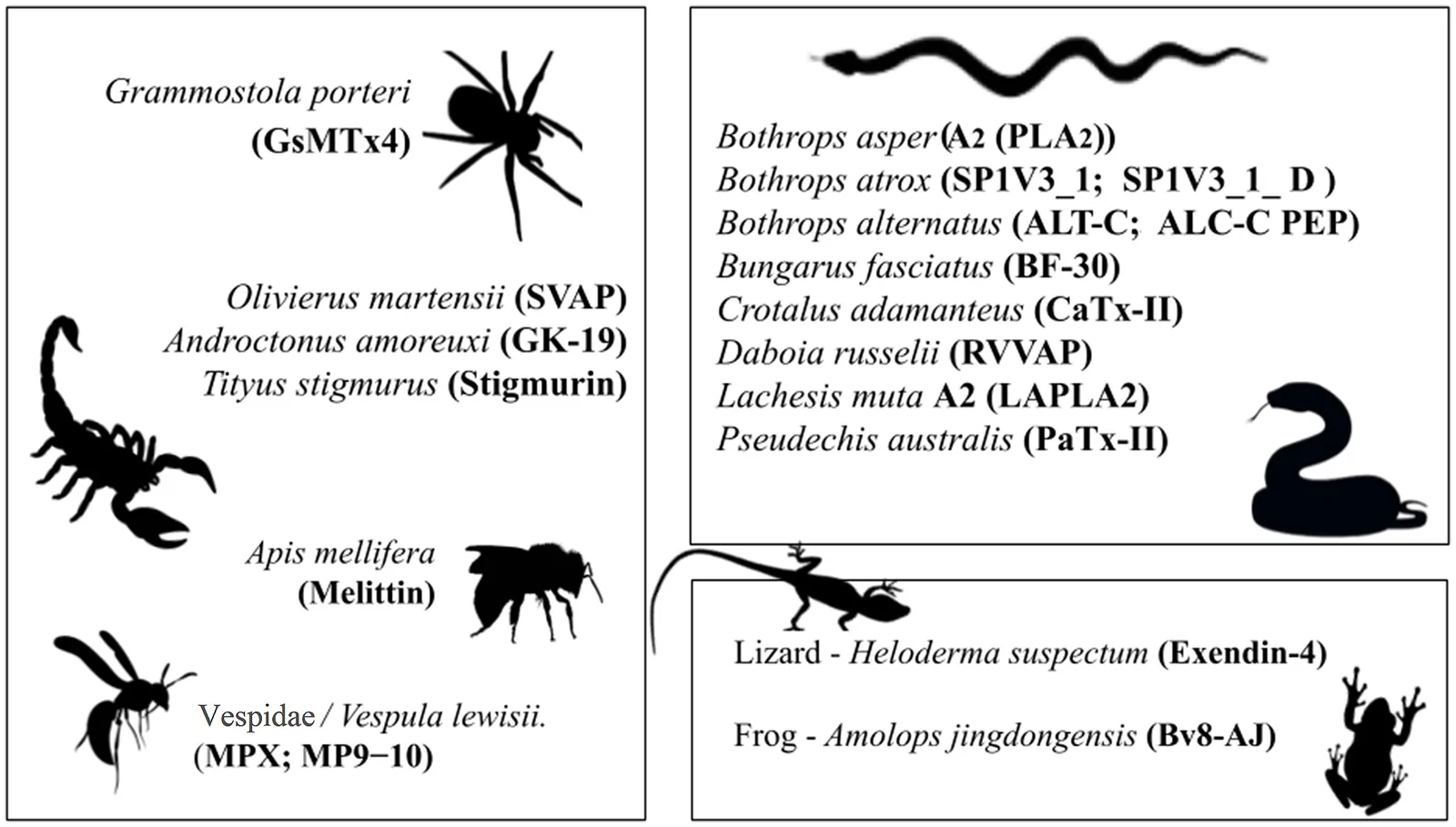
Diagram showing the bioactive peptides identified in this review and their respective animal origin. Animal species from which each peptide was first identified are shown in italics, and peptide names are shown in bold.

All included studies chose murine as the animal model for the search equation. Of the 19 articles, the most commonly used models were the Sprague-Dawley rat^17^^,^^19^^,^^24^^,^^27^ and BALB/c mice,^16^^,^^22^^,^^25^^,^^28^ each mentioned in 4 articles. Swiss mice,^11^^,^^14^^,^^21^ Kunming mice,^13^^,^^23^^,^^26^ and Wistar rats^5^^,^^15^^,^^16^ were mentioned in 3 articles each (Table 1).

**Table 1:** Data extract of the 19 selected articles.

Ref.	Pep.	Specie	Wound type, animal (*n*), and treatment	Outcomes
5	SP1V3_1 and SP1V3_1_ D	*Bothrops atrox*	✓ Septic wound animal models; ✓ 6 mm^2^ dorsal excision + *S. aureus* infection; ✓ WR ♀ and ♂ (6/group); ✓ 10 μM SP1V3_1 (solubilised in 1% glycerol base in PBS).	⊗ Toxicity in Mammalian cell lines and lymphocytes at 12.5-25 μM; ✓ Haemolytic activity of 20.42% at 25 μM SP1V3_1 concentration; ↓ Haemolytic and antimicrobial activity by SP1V3_1_D over SP1V3_1 at 6.25-100 μM; ✓ Only 17, 22% of open wound percentage at 11 days; ✓ Pharmacokinetic stability and anti-inflammatory effects (↓ IL-1, IL-6, IL-8, and TNF-ɑ).
11	A2 (LAPLA2)	*Lachesis muta*	✓ Aseptic wound animal models; ✓ 0,79 cm^2^ dorsal wound; ✓ SM ♂ (12/group); ✓ Topical treatment with 0.25% or 0.5% de LAPLA in water daily for 12 days.	✓ 93% of wound healed in 12 days; ↑ Distribution of mast cells with ↑ leucocytes recruitment, angiogenesis, colageno genesis, and reorganization of collagen fibers; ↑ Activity of the antioxidant enzymes SOD and CAT; ⊗ Toxicity to fibroblasts and keratinocytes (1.25-20 μg/mL); ↑ Keratinocytes proliferation at 10 μg/mL.
12	Exendin-4	*Heloderma suspectum*	✓ Aseptic wound animal models; ✓ Abrasion wounds (1.1 × 1.1 cm^2^); ✓ CD1 ♂ (6/group); ✓ Single intradermal injection 20 μL; ✓ Exe4 62 ng or Exendin-4 (9-39) 70 ng followed by 20 μL of Exe4 62 ng after 15 min.	↑ Vessel density, fibroblast migration and differentiation; ↓ Number of fibroblasts/myofibroblasts recovered in the wounds when both Exe4 and Exendin4 (9-39) were present; ✓ 90% of wound healed in 6 days with Exe4 62 ng.
13	Bv8-AJ	*Amolops jingdongensis*	✓ Aseptic wound animal models; ✓ Full thickness dorsal wound; ✓ KM ♀♂ (3/group); ✓ Bv8-AJ 100 μg/mL/day for 7 days.	✓70% of wound healed in 7 days; ↑ Inflammatory cell infiltration at day 3, but ↓ wound inflammation and scarring at day 7; ↑ Cell proliferation (keratinocytes and fibroblasts) only when they were cultured together; ↑ IL-1ɑ production in KC; ↑ Cell proliferation (keratinocytes and fibroblasts) only when they were cultured together; ↑ IL-1ɑ production in KC.
14	CaTx-II	*Crotalus adamanteus*	✓ Aseptic wound animal models; ✓ 2,6 cm^2^ dorsal wound + *S. aureus* infection; ✓ Pathogen free SM ♂ (6/group); ✓ 10 μg/kg single topical application.	↑ Wound healing, re-epithelialization, neovascularization and formation of collagen, fibroblasts and keratinocyte; ↑ MCP-1, FGF-basic, KC, and G-CSF after 16 days; ⊗ Cytotoxicity on tested human lung and skin fibroblast cells at high doses (15.6–500 μg/mL); ⊗ Toxic effects or kill mice up to 1–14 μg/kg, for 2 weeks.
15	ALT-C and ALT-C PEP	*Bothrops alternatus*	✓ Aseptic wound animal models; ✓ 4 mm^2^ dorsal excision; ✓ WR ♂ (5/group); ✓ Topical application of natrosol gel containing ALT-C 10, 60, 100 ng or ALT-C PEP 10, 20, 100 ng for 1, 3, 5, or 7 consecutive days.	✓ After day 3, ↑ blood vessel proliferation in all areas; ✓ Treatment with ALT-C 100 ng and ALT-C PEP 20 ng modulated VEGF, FGF-1, PDGF, TGF-β1, and IGF-1.
16	RVVAP	*Daboia russeli*	✓ Aseptic wound animal models; ✓ 6 mm^2^ dorsal excision; ✓ WR or BLC (6/group); ✓ 2.5 μM RVVAP; 35%-37% aloe vera in PBS 10000 μM; ✓ RVVAP combined with aloe vera*;* ✓ Topical application 2/day for 7 days in a cream-based formulation.	↑ Efficacy on wound healing combined with aloe vera; ↑ Wound healing with antibacterial effect; 80% of wound closed with 7 days; ⊗ Toxicity at 1000 μg/kg; ↓ Inflammatory cytokines (IL-1, IL-6, IL10, and TNF- α).
17	Exendin-4	*Heloderma suspectum*	✓ Aseptic wound diabetes induced animal models; ✓ 3,14 mm^2^ circular dorsal excision; ✓ SDR ♂ (6-8/group); ✓ Intraperitoneal injection; ✓ 500 μg/kg/day; ✓ Daily, for 14 days.	↑ Angiogenesis and vascularity via mobilization of EPCs; ↓ Haemorrhage; ↑ Dermal and epidermal regeneration; ⊗ Affect levels of serum glucose, triglyceride, and total cholesterol in diabetic rats; ↓ Proinflammatory status, superoxide anions and cytokine IL-6; ✓ TGF-β1/MMP-2 up regulation.
18	Exendin-4	*Heloderma suspectum*	✓ Aseptic wound diabetes induced animal models; ✓ 28 mm^2^ dorsal excision; ✓ Db/db or CBL ♂ (7-10/group); ✓ Topical 50 μL of 0.1 μM Ex-4 in 0.5% collagen-PBS alone or combined with intradermal injection ADSCs (2.5 × 10^5^ in PBS) injected around the wound daily for 13 days.	↑ Re-epithelialization when combined with ADSCs; ⊗ Affected Blood glucose and HbA1c levels; ↑ VEGF angiogenic effects on endothelial cells; ✓ADSCs have angiogenic effects on both endothelial cells and keratinocytes.
19	SVAP	*Olivierus martensii*	✓15 mm^2^ haemorrhagic ulceration; ✓SDR (7/group); ✓SVAP for 5 weeks.∗	↓ Inflammatory cytokines IL-1, IL-6, IL-8, and TNF-ɑ from 2nd to 5th week; ↓ Ulceration wounds at the 2nd week; ✓ Completely healed wounds at 3 weeks.
20	Melittin	*Apis mellifera*	✓ Septic wound animal models; ✓ *S. aureus* infection decorrent wound; ✓ CD1 ♂ (3/group); ✓ Subcutaneous administration; ✓ 10 μg of synthetic or purified melittin; ✓ Daily for 10 days.	⊗ Toxicity in human epithelial cells at 0.4 μg/mL and at 20000 μg/kg in mice; ✓ Synthetic melittin exhibited lower toxicity compared to the purified; ✓ Protective effects against intraperitoneal and skin wound infection; ↓ Diameters of the abscesses treated with melittin; ✓ 50% and 0% survival after 40 h in infected CD1 treated with 5000 μg/kg melittin and PBS respectively.
21	Stigmurin	*Tityus stigmurus*	✓ Septic wound animal models; ✓ 5 mm^2^ dorsal excision + *S. aureus* infection; ✓ SM ♂ (5/group); ✓ 20 μL daily topical Stigmurin (1 μg/μL) application over 7 days.	✓ 60% of wound closed with 7 days. ✓ Poor efficacy in ↓oxidative activity; ↓ Superoxide ion and hydroxyl radical; ↑ Wound healing process with antibacterial effect; 60% of wound closed with 7 days.
22	A_2_(PLA_2_)	*Bothrops asper*	✓ Aseptic wound animal models; ✓ 20 mm^2^ dorsal wound + *S. aureus* infection; ✓ BLC ♀ (5/group); ✓ Topical application; ✓ Gel mix 10000 μg/kg for 14 days.	↑ Healing wound process in 14 days; ↑ Formation of endothelial cells; ↑ Leukocyte infiltration and migration, MDC expression levels; ✓ Antimicrobial and bactericidal (MRSA *in vitro*).
23	GK-19	*Androctonus amoreuxi*	✓ Septic wound animal models; ✓0,5 cm^2^ dorsal scalded MRSA or *C. albicans* infection; ✓KM ♂ (5/group); ✓Topical treatment of 50 μM GK-19 twice a day for 12-16 days.	↑ Antimicrobial and healing effects combined with SSTIs induced both by MRSA and by *C*. *albicans;* ⊗ Cytotoxicity in Rat pulmonary cells, human keratinocytes, endothelial and kidney cells, at 1-10 μM ↓ Cell viability from 25 μM onward; ⊗ Mice toxicity with concentrations of 5 μM/kg and 25 μM/kg; ⊗ Haemolytic activity even at high concentrations (100 μM); ⊗ Induced drug resistance; ⊗ Observable tissue damage, apoptotic cells, necrosis, or inflammation; ✓ Potential for administration of GK-19 through the tail vein.
24	BF-30	*Bungarus fasciatus*	✓ Septic wound animal models; ✓ 4 cm^2^ dorsal scald injury +*P. aeruginosa* infection; ✓ SDR ♀ (?/group); ✓ 750, 3000, or 12000 μg/kg/day by intradermal injection;	↓ gram-negative bacteria at 2–32 μg/mL (MIC) ↓ gram-positive bacteria at 16–32 μg/mL (MIC) ↓ Colonization of *P. aeruginosa* at the wound area, lungs, and livers. ↓ Wound inflammation and scarring; ⊗ Induced drug resistance.
25	MPX	Vespidae	✓ Septic wound animal models; ✓Dorsal scratch + *S. aureus* infection; ✓CBL ♀ (10/group); ✓MPX ointment topical application twice a day for 7 days.∗	✓ Inhibition against *S. aureus* at 0.02 μM (MIC); ↓ Colonization at lungs and livers; ↓Inflammation; ↑ Wound healing with antibacterial effect and stability; ↑ Wound hair follicle gland.
26	MP9-10	*Vespula lewisii*	✓ Septic wound animal models; ✓8 mm^2^ dorsal excision + *S. aureus* infection; ✓KM ♀ (11/group); ✓50 μL daily topical application at 200 mM for 7 days.	⊗ Cytotoxicity in mouse macrophages cells, human keratinocyte and kidney cells at 4-32 μg/mL; ⊗ Haemolytic activity from 4 to 64 μg/mL; ✓Antibacterial activity against *E. coli* (MIC 4 μg/mL), *S. aureus* (MIC 4 μg/mL), *P. aeruginosa* (MIC 8 μg/mL) e *A. baumannii* (MIC 4 μg/mL); ↑ Wound healing with antibacterial effect on the third day; ↓ Inflammation.
27	GsMTx4	*Grammostola porteri*	✓ Aseptic wound animal models; ✓ 36 mm^2^ dorsal excision; ✓ SDR ♂ (6/group); ✓ Daily GsMTx4 (5 μM); ✓ Intradermal injections; ✓ For 14 days.	✓ GsMTx4 protects rats from stretch induced HS formation; ↓ Hyperaemia and scar elevation; ↓ Collagen density and α-SMA expression.
28	PaTx-II	*Pseudechis australis*	✓ Septic wound animal models; ✓ 20 mm^2^ dorsal excision + *S. aureus* infection; ✓ BLC ♀ (5/group); ✓ Topical application of PaTx-II for 4 days (500 μg/kg twice a day) by neutral gel.	⊗ Toxicity in human skin (HEPK), lung fibroblast (MRC-5), and macrophage (THP-1) up to 1000 μM; ↑ Cell proliferation; ↑ Vascularisation, re-epithelialization and type I collagen; ↓ Wound inflammation and scarring; ↓ Cytokines IL-1, IL-6, IL-10, and TNF-ɑ; ✓ Completely healed wound at 14 days.

Ref.: reference; Pep.: peptides; WR: Wistar rats; PBS: phosphate-buffered saline; LAPLA2: acidic phospholipase A2; SM: Swiss mice; SOD: superoxide dismutase; CAT: catalase; CD1: CD1 mouse; Exe4: Exendin-4; KM: Kunming mice; IL: interleukin; KC: keratinocyte chemoattractant; MCP-1: monocyte chemoattractant protein 1; FGF: fibroblast growth factor; G-CSF: granulocyte colony-stimulating factor; ALT-C: Alternagin-C; VEGF: vascular endothelial growth factor; PDGF: platelet-derived growth factor; TGF-β1: transforming growth factor-beta; IGF-1: insulin-like growth factor-1; RVVAP: Russell's viper venom-associated peptide; TNF-α: tumor necrosis factor-alpha; SDR: Sprague Dawley rats; EPCs: endothelial progenitor cells; MMP: matrix metalloproteinase; ADSCs: adipose-derived stem cells; SVAP: scorpion venom active polypeptide; BLC: BALB/c mouse; MDC: macrophage-derived chemokine; MRSA: methicillin-resistant *Staphylococcus aureus*; SSTIs: skin and soft tissue infections; MIC: minimum inhibitory concentration; MPX: Mastoparan; CBL: C57BL/6 mouse; HbA1c: glycated hemoglobin; α-SMA: alpha-smooth muscle actin; HS: hypertrophic scar; HEPK: human epidermal keratinocytes; MRC-5: lung fibroblasts; THP-1: macrophages.

♂: male; ♀: female; ✓: confirmed/present; ↑: increased; ↓: decreased; ⊗: did not induce; ∗: no concentrations disclosed.

Most of the wounds were full-thickness dorsal excisions performed with a biopsy punch, with the area varying depending on the size of the animal model. Care was taken not to injure the muscle tissue of the animals studied, as the studies were mainly aimed at assessing skin healing (wound healing). All wounds were created under sterile conditions. Nevertheless, 42.1% (8/19) of the models were inoculated with selected bacteria after excision to induce inflammation and infection.^5^^,^^20^^,^^21^^,^^23^, ^24^, ^25^, ^26^^,^^28^

### 3.2 Activity outcome

It has been shown that peptides accelerate wound closure. For example, exendin-4 (from *Heloderma suspectum*) led to 90% wound healing in CD1 mice within 6 days.^12^ Bv8-AJ (from Amolops jingdongensis) led to 70% healing within 7 days in Kunming mice.^13^ Acidic phospholipase A2 (LAPLA2) (from *Lachesis muta*) achieved 93% healing in Swiss mice within 12 days.^11^ In infected wound models, CaTx-II (from *Crotalus adamanteus*) and A_2_ (PLA_2_) (from *Bothrops asper*) improved wound healing.^14.22^ Peptides such as stigmurine (from *Tityus stigmurus*) and PaTx-II (from *Pseudechis australis*) also promoted healing in infected wounds, with PaTx-II leading to complete wound closure within a 14 days.^21^^,^^28^

Peptides such as exendin-4 (in Sprague Dawley rats (SDR) diabetic rats) increased angiogenesis, vascularisation, and dermal and epidermal regeneration.^17^ Other peptides, including CaTx-II, promoted re-epithelialization, neovascularisation, and the formation of collagen, fibroblasts, and keratinocytes.^14^ PaTx-II also induced vascularisation, re-epithelialization, and the production of type I collagen.^28^ Bv8-AJ and LAPLA2 enhanced keratinocyte proliferation, while Bv8-AJ also stimulated fibroblast proliferation.^11^^,^^13^ PaTx-II likewise promoted cellular proliferation.^28^

Treatment with Alternagin-C and ALT-C PEP (synthetic peptide derived from Alternagin-C) from *Bothrops alternatus* modulated the levels of vascular endothelial growth factor (VEGF), fibroblast growth factor (FGF)-1, fibroblast growth factor (PDGF), transforming growth factor-beta (TGF-β1), and insulin-like growth factor-1.^15^ Exendin-4, in SDR diabetic rats, also resulted in the upregulation of TGF-β1/matrix metalloproteinase (MMP)-2.^17^

GsMTx4 (from *Grammostola porteri*) reduced hyperaemia and scar elevation, as well as collagen density and α-SMA expression.^27^ Bv8-AJ reduced wound inflammation and promoted healing within 7 days.^13^ Russell's viper venom-associated peptide (RVVAP) (from *Daboia russelii*), when combined with aloe vera, decreased inflammatory cytokines (IL-1, IL-6, IL-10, and tumor necrosis factor-alpha (TNF-α)).^16^ Scorpion venom active peptide (from Olivierus martensii) reduced inflammatory cytokines (IL-1, IL-6, IL-8, and TNF-α) in ulcerated wounds.^19^ PaTx-II reduced both inflammation and wound healing time, as well as levels of IL-1, IL-6, IL-10, and TNF-α.^28^ BF-30 (from *Bungarus fasciatus*) reduced inflammation and accelerated wound repair.^24^ Mastoparan (from Vespa) and MP9-10 (from *Vespula lewisii*) also demonstrated anti-inflammatory effects.^25^^,^^26^

Several peptides have demonstrated antimicrobial activity against bacteria such as *Staphylococcus aureus*, *Pseudomonas aeruginosa*, *Escherichia coli*, and *Candida albicans*. A_2_(PLA_2_) from *Bothrops asper* exhibited *in vitro* antimicrobial and bactericidal effects.^22^ Stigmurin and RVVAP demonstrated antibacterial activity.^16^^,^^21^ GK-19 (from *Androctonus amoreuxi*) displayed both antimicrobial and wound-healing effects in infections caused by methicillin-resistant *Staphylococcus aureus* and *C. albicans*.^23^ BF-30 inhibited both Gram-negative and Gram-positive bacteria and reduced *Pseudomonas aeruginosa* colonization.^24^ Mastoparan inhibited *Staphylococcus aureus*.^25^ MP9-10 showed antibacterial activity against *Escherichia coli*, *Staphylococcus aureus*, *Pseudomonas aeruginosa*, and *Acinetobacter baumannii*.^26^

### 3.3 Toxicity outcome

Some peptides exhibited toxicity at elevated doses. LAPLA2 was found to be toxic to fibroblasts and keratinocytes.^11^ CaTx-II demonstrated cytotoxicity in human pulmonary and dermal fibroblasts at high concentrations.^14^ Melittin (from *Apis mellifera*) was toxic to human epithelial cells.^20^ PaTx-II showed toxicity in human skin cells (HEPK), pulmonary fibroblasts (MRC-5), and macrophages (THP-1).^28^ SP1V3_1 (from *Bothrops atrox*) was toxic to mammalian cell lines and lymphocytes.^5^ GK-19 exhibited cytotoxicity in rat lung cells, human keratinocytes, endothelial cells, and renal cells.^23^ MP9-10 was cytotoxic to murine macrophages, human keratinocytes, and renal cells.^26^

CaTx-II proved to be toxic or lethal to mice at doses ranging from 1 to 14 μg/kg.^14^ RVVAP exhibited toxicity at 1 mg/kg.^16^ Melittin was toxic to mice at a dose of 20 mg/kg.^20^ GK-19 demonstrated toxicity in mice at concentrations of 5 μM/kg and 25 μM/kg.^23^

GK-19 and BF-30 were observed to induce drug resistance,^23^^,^^24^ while SP1V3_1 and GK-19 displayed haemolytic activity.^5^^,^^23^ MP9-10 also exhibited haemolytic activity.^26^ Furthermore, Exendin-4 (in SDR diabetic rats) affected serum glucose levels, triglycerides, and total cholesterol.^17^

### 3.4 Risk of bias evaluation

Based on the SYRCLE's RoB tool analysis,^9^ the comparison studies by different authors reveal varying levels of methodological rigor and reporting quality, with some demonstrating a lower risk of bias than others (Fig. 3).

**Fig. 3:**
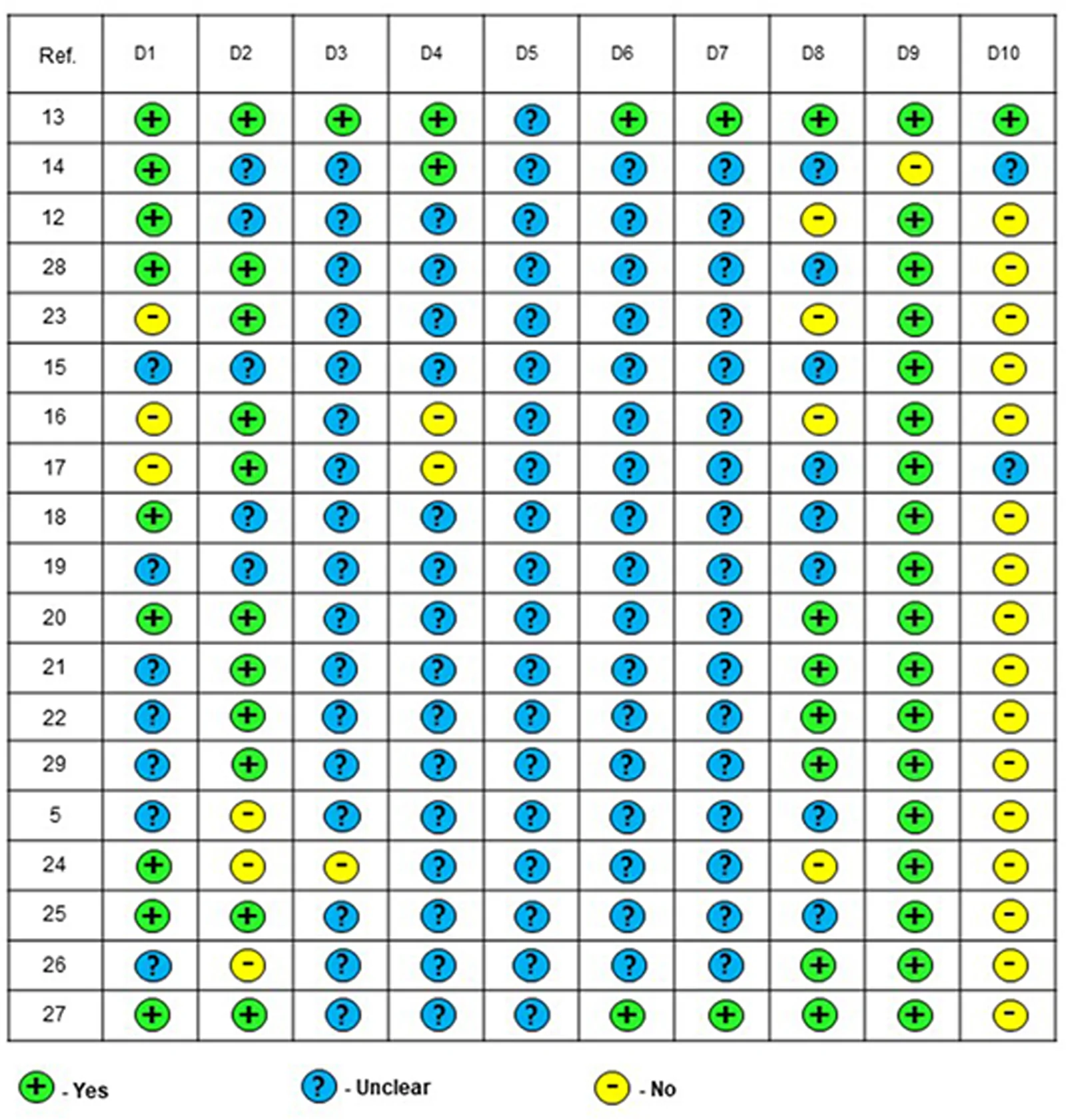
SYRCLE's RoB tool for assessing risk of bias in animal studies. Type of bias/Domain: D1: selection bias/sequence generation; D2: selection bias/baseline characteristics; D3: selection bias/allocation concealment; D4: performance bias/random housing; D5: performance bias/blinding; D6: detection bias/random outcome assessment; D7: detection bias/blinding; D8: attrition bias/incomplete outcome data; D9: reporting bias/selective outcome reporting; D10: other sources of bias SYRCLE tool.^10^

Two studies stand out with a consistently low risk of bias. This suggests a high level of methodological quality, with adequate reporting of randomization, blinding, and handling of outcome data.^12^^,^^26^ Some studies consistently show a high risk of bias in several crucial domains. This presents a potential risk for allocation concealment, random housing, and blinding of caregivers.

Other studies present a more mixed picture, with a low risk of bias in some areas but a substantial number of “unclear” ratings, particularly in domains related to blinding and random selection for outcome assessment. This suggests that while these studies may have followed some good practices, the reporting of their methods was insufficient to allow for a confident assessment of bias.

## 4 Discussion

Peptides isolated from the venoms of various species, including snakes, scorpions, lizards, frogs, wasps, bees, and spiders, exhibit a broad spectrum of therapeutic activities, such as antimicrobial effects, stimulation of cell proliferation, induction of neovascularization, and modulation of the inflammatory response.^29^ These activities are important in all 4 phases of the wound healing process, particularly during the hemostatic, inflammatory, and proliferative phases.^30^

Compared to agents traditionally used in clinical wound care, such as antimicrobials, anti-inflammatory drugs, debridement agents or isolated growth factors (e.g., epidermal growth factor, VEGF, or PDGF), peptides derived from venom are characterised by their ability to act synergistically in multiple phases of tissue repair.^6^^,^^29^^,^^31^

### 4.1 Peptides in the haemostasis phase

The first phase of wound healing, known as haemostasis, is responsible for controlling bleeding and preparing the tissue for the subsequent phases of repair.^2^^,^^32^ In this context, peptides have demonstrated, *in vivo*, the ability to modulate vascular permeability and thus promoting the extravasation of coagulation factors from the blood vessels into the injured tissue.^33^ In addition, they promote a pro-coagulant state through the induction of pro-inflammatory cytokines such as IL-1, TNF-α, and IL-6, which indirectly supports haemorrhage control by activating the coagulation cascade.^4^^,^^34^

Tissue injuries not only trigger the coagulation cascade but also often a nociceptive reaction (Fig. 4). This nociceptive response plays an adaptive role by heightening awareness of the injury and promoting protection of the affected area. However, it can also have negative effects by impairing local function and having a negative impact on psychological and social well-being.^35^

**Fig. 4:**
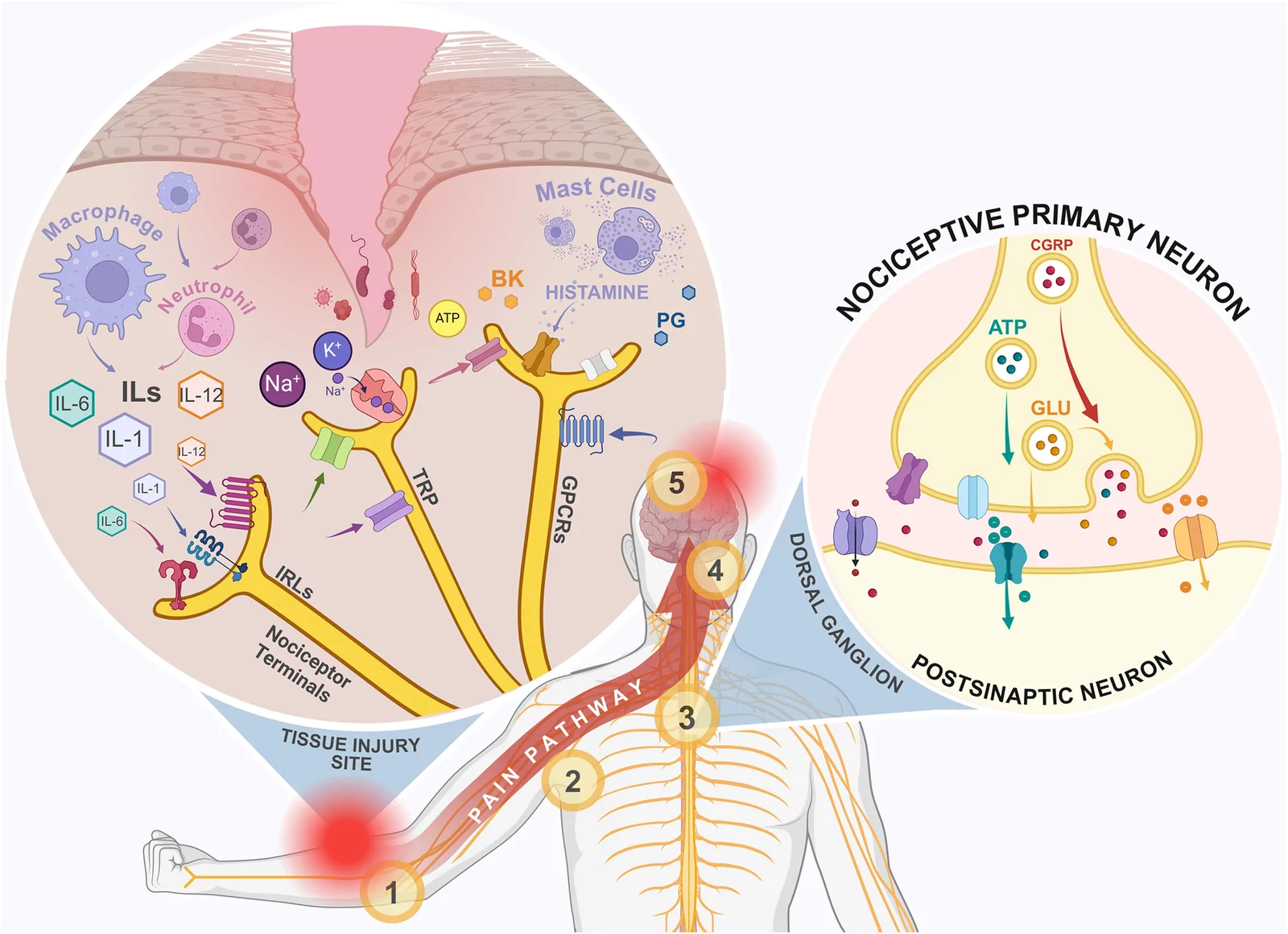
Transduction pathway of noxious stimuli. The inflammatory process initiated by microbial pathogens in the wounded tissue causes release of multiple components such as ions (Na+, K+), ATP molecules, chemical mediators (BK, PG, histamine), and pro-inflammatory ILs such as IL-1, IL-12 and IL-6. Each nociceptor terminal belongs to a category such as: IRLs, TRP, and GPCRs. After transducing the stimuli through the peripheral nerve fibre, the connection is mediated by neurotransmitters that induce synapses, allowing the brain to recognise the nociceptive signal. ATP: adenosine triphosphate; BK: bradykinin; PG: prostaglandins; IL: interleukin; IRLs: interleukin receptors; TRP: transient receptor potential channel; GPCRs: metabotropic G-protein coupled receptors; GLU: glutamic acid.

Peptide-induced sensitization to nociception at the site of injury (hyperalgesia) occurs via several mechanisms, including the release of cytokines, chemokines, prostaglandins, serotonin and histamine, all of which modulate nociceptive signaling and sensitise peripheral nerve fibres.^36^ This process may also include modulation of TRPV1 (Transient Receptor Potential Vanilloid 1) and TRPA1 (Transient Receptor Potential Ankyrin 1) receptors, the bypass of μ-opioid receptors or changes in vascular permeability, contributing to the oedema associated with the inflammatory response.^36^^,^^37^

As far as the pharmacological principles are concerned, animal venom peptides could act as a novel approach to nociception management in multiple types of patients, where traditional analgesics are associated with risks of tolerance, toxicity, and dependence.^37^^,^^38^ The diverse mechanisms of action of these peptides are associated with their role in modulating nociceptive signals. Phospholipase-related rich polypeptides (A2, A2PLA2), such as those found in Ants Venoms^39^, ^40^, ^41^ and honeybee (Apis mellifera) venom,^42^^,^^43^ act by regulating the release of inflammatory mediators from peripheral tissues via TRPA1 (Transient Receptor Potential Ankyrin 1) receptors, thereby affecting the peripheral nervous system. Although these types of venom are traditionally pro-inflammatory, melittin has been studied for its complex effects on both the central and the peripheral nervous system, exhibiting antinociceptive properties under specific conditions.^42^ Another centrally acting analgesic, reported to be significant potent, is Polybia occidentalis wasp venom, which has been described as active in via the modulation of G-protein-coupled B2 bradykinin receptors.^44^

Despite animal venom peptides showing potential for nociception relief, there remains a lack of continuity in experiments combining this property with the wound healing effects discussed in this article. Also, it is important to emphasise that pain cannot be reduced only by neural influence.^35^ There are psychological, biological, and social factors, which must all be considered.

### 4.2 Peptides in the inflammatory phase

After haemostasis and subsequent breach of the body's primary protective barrier, microbial invasion becomes a critical problem as it can significantly worsen the wound condition.^45^ Infected wounds pose a significant challenge to the healing process as they can contribute to chronic inflammation, delay tissue repair, and increase the risk of serious complications such as sepsis.^30^^,^^46^ In this context, infection control in a clinical setting is crucial, as uncontrolled infection may lead to amputation or result in prolonged hospitalization.

Effective treatment of wound infections is therefore crucial to ensure proper healing and prevent further complications. However, given the global rise in microbial resistance, additional antimicrobial measures have become urgently necessary. This occurs mainly due to the overuse and misuse of antibiotics, which have led to the spread of multidrug-resistant strains of bacteria that complicate infection management and increase morbidity, healthcare costs, and mortality.^47^^,^^48^

In this scenario, antimicrobial peptides (AMPs) have emerged as promising alternatives, as they possess a broad antimicrobial spectrum of activity and different mechanisms of action that minimise the propensity for resistance development.^45^^,^^47^ Their multifunctional role in modulating inflammation and promoting healing makes AMPs candidates for next-generation wound therapies.^49^^,^^50^ AMPs interact with negatively charged components of microbial membranes, which leads to pore formation, membrane disruption, and ultimately, microbial cells death.^49^^,^^50^ This physical mechanism reduces the likelihood of resistance developing compared to target-specif conventional antibiotics.^50^

At the same time, certain peptides play a role in modulating the inflammatory response by activating mast cells and macrophages, which in turn induce the release of pro-inflammatory cytokines and recruit neutrophils and monocytes, thereby enhancing the activation of the innate immune system.^34^ The inflammatory process is characterised by the classic manifestation of the 5 cardinal signs: redness, heat, swelling, pain, and in severe cases, loss of function.^51^ Prolonged inflammation is detrimental to wound healing, as observed in chronic wounds of diabetic patients.^17^ In addition, prolonged exposure to pathogens during this phase increases the risk of secondary infection, systemic inflammation and clinical complications such as thrombosis, hypotension, cachexia, and sepsis.^5^ Therefore, appropriate regulation of the inflammatory response is necessary to prevent complications and promote optimal tissue regeneration. Several studies have shown that peptides from animal venoms exert anti-inflammatory effects.^37^ In 9 experimental studies investigated, these animal venom-derived peptides outperformed the control group in effectively modulating inflammation, emphasising their therapeutic potential for wound healing.

IL-1, IL-6, and IL-8 play an active role in the inflammatory response.^34^^,^^51^ These interleukins are responsible for increasing inflammation by stimulating the production of leukocytes and promoting the expression of adhesion molecules that are important for the migration of leukocytes.^51^ They also mediate systemic symptoms associated with acute inflammation. The downregulation of these cytokines by peptides has been associated with reduced tissue regeneration time, reduced intensity of inflammatory symptoms, and prevention of scarring.^52^ Therefore, suppression of these pro-inflammatory cytokines is crucial for effective wound healing and modulation of the inflammatory phase.

Another important inflammatory mediator is TNF-α, which plays a dual role: it contributes to the acute inflammatory response and enhances host defense against bacterial infections by activating Toll-like receptors in response to bacterial cell wall components.^53^^,^^54^ However, overexpression of TNF-α is associated with chronic inflammatory diseases and septic shock.^51^ The downregulation of TNF-α observed in several studies with venom-derived peptides represents a favourable outcome in the control of inflammation.^5^^,^^14^^,^^16^^,^^28^

Not all peptides have exclusively anti-inflammatory effects. Compounds such as Bv8-AJ^13^ and A2(PLA_2_)^11^ have demonstrated the ability to stimulate inflammation, which can have a positive clinical effect in especific contexts. For example, the upregulation of IL-1 can promote the proliferation of keratinocytes and fibroblasts, which contributes to a improved re-epithelialization and wound closure.^13^ A_2_(PLA_2_) has also been associated with keratinocyte hyperplasia,^11^ which facilitates the transition from inflammatory phase to the proliferative phase of wound healing.

### 4.3 Peptides in the proliferative phase

After the inflammatory phase of wound healing, the proliferative phase begins, which is characterised by the release of growth factors, cytokines and other mediators by inflammatory cells. This phase is crucial for tissue repair and involves the proliferation of fibroblasts, keratinocytes, and endothelial cells, as well as the deposition of extracellular matrix and the formation of new blood vessels.^2^^,^^4^

Angiogenesis, the formation of new blood vessels, is a key process in this phase that ensures an adequate supply of oxygen and nutrients to the regenerating tissue.^2^^,^^32^ Studies have shown an association between angiogenesis and wound healing outcomes, while insufficient angiogenesis can lead to chronic ischaemic wounds and an increased risk of tissue necrosis.^55^

Neovascularisation is based on the activation, proliferation, and migration of resident microvascular endothelial cells, processes controlled by several pro-angiogenic factors.^4^ The well-characterised mediators include VEGF, FGF, PDGF, epidermal growth factor, and TGF-β.^4^^,^^34^ These factors act synergistically to stimulate cell proliferation, matrix remodelling and the release of other pro-angiogenic mediators. These growth factors are positively regulated in the wound fluid of experimental models such as pigs, mice, and humans, suggesting that their presence is a common and essential response to skin injury.

In addition to endogenous mediators, there is increasing evidence that peptides from animal toxins may also play a role in the proliferative phase.^45^ These peptides promote the proliferation of fibroblasts, keratinocytes, and thus accelerate re-epithelialization and wound closure.^17^^,^^56^ They also induce angiogenesis and thus improve the supply of oxygen and nutrients to the regenerating tissue.^33^ In addition, they promote collagen synthesis and organization, thereby strengthening scar tissue, regulating MMPs, and TGF-β, contributing to proper remodelling of the extracellular matrix.^1^^,^^2^^,^^4^

For example, exendin-4, a peptide derived from lizard venom, has been shown to promote vascularisation and regeneration of skin and epidermis, while reducing bleeding. In combination with adipose-derived stem cells, it increased the expression of VEGFR-2 in the papillary epidermal layer of diabetic wounds. Markers such as Ki-67 and TGF-β1 were also upregulated in the subepidermal layer, indicating increased proliferative activity.

Other peptides, such as LAPLA2 and PaTx-II, derived from snake venoms, have shown abilities to positively regulate pro-angiogenic factors such as VEGF, FGF, and TGF-β, thereby increasing vascular density and promoting cell migration. These effects are attributed to their molecular structures that facilitate interactions with specific receptors, often through unique peptide sequences or stable conformations maintained by disulfide bonds.^29^^,^^33^^,^^57^ For example, peptides isolated from frogs have been shown to activate the IL-1 signalling pathway, thereby promoting fibroblast and keratinocyte proliferation.^58^ Similarly, bee venom has shown efficacy in preventing collagen degradation by inhibiting matrix metalloproteinases (MMP-1 and MMP-3), likely due to its antioxidant properties.^33^^,^^59^

In addition, these peptides modulate the inflammatory environment by promoting the recruitment of leukocytes, which in turn stimulate local collagen synthesis through the release of growth factors.^22^ Although the exact cellular mechanisms underlying these effects are not yet fully understood, it is known that venom-derived peptides can mimic hormones to specific receptors to activate intracellular signalling pathways such as exendin-4, which exerts its effect via the GLP-1 receptor.^29^^,^^33^^,^^57^

### 4.4 Challenges to clinical translation

Although the preclinical results on venom-derived peptides for wound healing are promising, their successful translation into clinical practice faces several significant obstacles.^6^

The first point concerns their industrial production. The large-scale production of venom-derived peptides for clinical use represents a major obstacle. The extraction of natural venom is often inefficient, expensive, ethically challenging and requires specialised facilities.^33^ Therefore, recombinant DNA techniques (e.g., bacterial, yeast, insect, or mammalian cells) can be used, which, like solid phase synthesis, are complex and costly.^60^ In addition, purification processes for pharmaceutical-grade peptides are demanding and expensive, increasing overall production costs and limiting accessibility.

Another important issue is their stability. Peptides are inherently unstable as they are susceptible to enzymatic degradation (proteolysis), aggregation, and denaturation.^61^ This instability can significantly affect their shelf life and bioavailability, especially when administered topically or systemically for wound healing. The development of stable formulations that protect the peptide from degradation while ensuring its sustained release at the wound site is crucial. This often requires innovative delivery systems such as hydrogels, nanoparticles, liposomes, or microneedle patches. Each of these systems requires extensive research and development to ensure biocompatibility, controlled release kinetics and effective penetration into the target tissue while maintaining the therapeutic activity of the peptide.

This study is subject to several potential biases. Selection bias may arise primarily from the lack of randomization in animal studies. Performance and detection biases are likely, as investigators are often not blinded. Reporting bias is also a concern, as studies with positive results are more likely to be published, which can lead to an overestimation of the actual effect. Bias in the results may arise from the unequal loss of animals between the treated and control groups.

Additional factors contributing to bias and heterogeneity include the considerable differences in the animal models used, experimental designs, outcome measures, and animal treatment procedures. In addition, there is considerable heterogeneity in the peptides tested, including differences in chemical structure, formulations that affect bioavailability, stability, and purity, all of which may influence results. These limitations contribute to both systematic bias and increased heterogeneity in the reported results.

In conclusion, animal venom, often perceived as a harmful substance, is in fact a rich source of molecules with various biological properties that are of interest to science and pharmacy. Among these properties, the ability to promote wound healing has been documented by numerous scientific reports, including this article. These biocomponents contained in animal venom can modulate different phases of the complex wound healing process, from haemostasis and inflammation to cell proliferation and tissue remodelling. This versatility provides a basis for the development of new topical biopharmaceuticals with therapeutic potential for the treatment of hard-to-heal wounds.

## CRediT authorship contribution statement

**Alessandra Silva Teles:** Writing – original draft, Methodology, Formal analysis, Data curation, Conceptualization. **Thais Lima:** Writing – original draft, Methodology, Investigation, Formal analysis, Data curation, Conceptualization. **Andréa Gonçalves de Melo:** Writing – original draft, Methodology. **William Gustavo Lima:** Writing – review & editing. **Lívia Cristina de Paiva Gardoni:** Writing – original draft, Conceptualization. **Israel Júnior Borges do Nascimento:** Writing – review & editing. **Julio Cesar Moreira Brito:** Writing – review & editing, Writing – original draft, Validation, Supervision, Resources, Project administration, Methodology, Investigation, Funding acquisition, Formal analysis, Data curation, Conceptualization.

## Ethical statement

Not applicable.

## Funding

None.

## Declaration of competing interest

All authors report that they do not have any conflicts of interest.
