# Supplementary material for: Toxins turned into medicines: A systematic review of peptide animal-derived venom in wound treatments
Source: Chin J Traumatol. 2026 Apr 25;29(5):391–401. doi: 10.1016/j.cjtee.2025.08.006 (PMC13654360; doi:10.1016/j.cjtee.2025.08.006)
Supplement: Supplementary file 1 [file mmc1.docx]

### SUPPLEMENTARY FILE

**Toxins turned into medicines: A systematic review of peptide animal-derived venom in wound treatments**

Alessandra Silva Teles^a,e^, Thais Lima^b,e^, Andréa Gonçalves de Melo^b^, William Gustavo Lima^c^, Lívia Cristina de Paiva Gardoni^b^, Israel Júnior Borges do Nascimento^d^, Julio Cesar Moreira Brito^b,*^,

julio.brito@funed.mg.gov.br

^a^Universidade Federal de Minas Gerais, Belo Horizonte, Minas Gerais, Brazil

^b^Fundação Ezequiel Dias (FUNED), Belo Horizonte, Minas Gerais, Brazil

^c^Instituto de Ensino e Pesquisa, Santa Casa, Belo Horizonte, Minas Gerais, Brazil

^d^Division of Country Health Policies and Systems (CPS), World Health Organization, Regional Office for Europe, Copenhagen, 2100, Denmark

^*^Corresponding author

^e^These authors contributed equally to this work.

**Details of the search strategy - July 07, 2025**

**Search in Pubmed:**

#1 "Peptides"[Title/Abstract] OR "Peptide"[Title/Abstract] OR "Polypeptide"[Title/Abstract] OR "Polypeptides"[Title/Abstract]

#2 "Venoms"[Title/Abstract] OR "Venom"[Title/Abstract]

#3 "wound"[Title/Abstract] OR "injury"[Title/Abstract] OR "injuries"[Title/Abstract] OR "wounds"[Title/Abstract] OR "wounded"[Title/Abstract] OR "wounding"[Title/Abstract] OR "woundings"[Title/Abstract] OR "sore"[Title/Abstract] OR "sores"[Title/Abstract] OR "hurt"[Title/Abstract] OR "hurts"[Title/Abstract] OR "damage"[Title/Abstract] OR "harmed"[Title/Abstract] OR "bruised"[Title/Abstract] OR "battered"[Title/Abstract] OR "cicatrization"[Title/Abstract]

#4 "animal"[Title/Abstract] OR "animals"[Title/Abstract] OR "rat"[Title/Abstract] OR "rats"[Title/Abstract] OR "mice"[Title/Abstract] OR "mouse"[Title/Abstract] OR "rabbit"[Title/Abstract] OR "murine"[Title/Abstract] OR "in vivo"[Title/Abstract]

**Search in Scopus:**

TITLE-ABS-KEY ( "Peptides" OR "Peptide" OR "Polypeptide" OR "Polypeptides" ) AND TITLE-ABS-KEY ( "Venoms" OR "Venom" ) AND TITLE-ABS-KEY ( "wound" OR "injury" OR "injuries" OR "wounds" OR "wounded" OR "wounding" OR "woundings" OR "sore" OR "sores" OR "hurt" OR "hurts" OR "damage" OR "harmed" OR "bruised" OR "battered" OR "cicatrization" ) AND TITLE-ABS-KEY ( "animal" OR "animals" OR "rat" OR "rats" OR "mice" OR "mouse" OR "rabbit" OR "murine" OR "in vivo" )

**Search in EMBASE:**

('peptides':ti,ab,kw OR 'peptide':ti,ab,kw OR 'polypeptide':ti,ab,kw OR 'polypeptides':ti,ab,kw) AND ('venoms':ti,ab,kw OR 'venom':ti,ab,kw) AND ('wound':ti,ab,kw OR 'injury':ti,ab,kw OR 'injuries':ti,ab,kw OR 'wounds':ti,ab,kw OR 'wounded':ti,ab,kw OR 'wounding':ti,ab,kw OR 'woundings':ti,ab,kw OR 'sore':ti,ab,kw OR 'sores':ti,ab,kw OR 'hurt':ti,ab,kw OR 'hurts':ti,ab,kw OR 'damage':ti,ab,kw OR 'harmed':ti,ab,kw OR 'bruised':ti,ab,kw OR 'battered':ti,ab,kw OR 'cicatrization':ti,ab,kw) AND ('animal':ti,ab,kw OR 'animals':ti,ab,kw OR 'rat':ti,ab,kw OR 'rats':ti,ab,kw OR 'mice':ti,ab,kw OR 'mouse':ti,ab,kw OR 'rabit':ti,ab,kw OR 'murine':ti,ab,kw)

**BVS:**

("Peptides" OR "Peptide" OR "Polypeptide" OR "Polypeptides" ) AND ("Venoms" OR "Venom") AND ("wound" OR "injury" OR "injuries" OR "wounds" OR "wounded" OR "wounding" OR "woundings" OR "sore" OR "sores" OR "hurt" OR "hurts" OR "damage" OR "harmed" OR "bruised" OR "battered" OR "cicatrization") AND ("animal" OR "animals" OR "rat" OR "rats" OR "mice" OR "mouse" OR "rabbit" OR "murine" OR "in vivo")
